# Paralysis in Children

**Published:** 1908-07-18

**Authors:** James Taylor

**Affiliations:** Physician to the National Hospital for the Paralysed and Epileptic, to the North-Eastern Hospital for Children, and to the Royal London Ophthalmic Hospital.


### Hospital Clinics.

### PARALYSIS IN CHILDREN.

By JAMES TAYLOR, M.A., M.D., F.R.C.P., Physician to the National Hospital for the Paralysed and Epileptic, to the North-Eastern Hospital for Children, and to the Royal London Ophthalmic Hospital.

(Abstract of a lecture delivered at the National Hospital, Queen Square.]

Before dealing briefly with the commoner forms of paralysis in children, I will show a few cases which illustrate the appearances of patients suffering from some of them.

### Some Actual Cases.

This child, for instance, who has just come in is a typical example of one form of paralysis as it occurs in children. He was perfectly well until one morning he complained that his leg ached. In the afternoon he was unable to walk, and the limb became progressively weaker, until, in a few days, it was completely paralysed. The disease from which he suffered was, of course, anterior poliomyelitis. Infantile paralysis is not a very good name for this condition, for, though it is much commoner in children, the disease does also affect adults at all times of life.

Here is another child suffering from a form of cerebral diplegia. There is little doubt that this is the result of encephalitis and vascular disease due to congenital syphilis. In this third case you will see there is no actual paralysis, but merely backwardness, lethargy and weakness. The child is, in fact, a cretin, and it has already begun rapidly to improve under treatment with extract of thyroid gland.

I had a similar case a few years ago of a cretinous child, which, although it had reached four and a half years of age, had not cut any of its milk teeth. Its hair was thin and scanty, its belly enlarged with protruding umbilicus, and it was of very low intelligence. Within two months the hair had grown quite thick, the teeth were rapidly cut, the abdomen ceased to bulge, and a great change in the mental character supervened. Since then the child has been fed regularly with thyroid extract, and is now a fairly healthy girl of fifteen or so, though she is still three or four years younger than that mentally. The only sign of relapse was exhibited when the medication with thyroid extract was omitted for three weeks.

With this next child there is a history of a fit followed by weakness of the right arm and leg. He is still subject to fits, and though not absolutely paralysed is still very weak on the right side. He is almost completely aphasic, and the only word he can say is one which we may suppose is among the first he learnt in infancy before his fit: it is the one word "damn."

### Pseudo-hypertrophic Paralysis.

In this child whom you now see there is great difficulty in standing, though the leg muscles look large, even larger than usual. The reason is a pseudo-hypertrophic paralysis of the calf muscles, and contracture of the hamstrings. This is an example of one of the varieties of myopathy.

This girl has a left hemiplegia of long standing, ever since infancy indeed. Probably in her case there was some process affecting the cells of the cortex of the brain of a similar nature to that which occurs in the spinal cord in cases of anterior poliomyelitis.

I now propose to enumerate shortly the various forms of paralysis in children, and there are certain good reasons why they should be separately considered from those of adults. Thus the great majority of cases of paralysis in adult life are associated with vascular disease, which in children is extremely rare. Another point we ought to remember is that the structures affected in children are still growing, so that deformities are often produced quite different to those that might occur under similar circumstances in adult life. Thus shortening of a limb, for example, is a not infrequent result of paralysis in childhood.

It is convenient to consider three groups among the paralytic diseases of childhood. These are respectively the cerebral, the spinal, and the myopathic.

### Cerebral Palsies.

Birth palsy is a convenient term for those cases of paralysis that exist from the very moment of birth. They are of different degrees and varieties, but the most common is a spastic diplegia of the legs usually known as congenital diplegia. There can be little doubt that one cause of this is hæmorrhage under the vertex of the skull in difficult labour, and there are several significant facts in favour of that view. A large number of the cases are in first-born children, who are much more exposed to difficulty and injury in parturition; and also many more males than females are affected by this diplegia. Moreover, actual examination has shown that still-birth is sometimes due to a further degree of this vertical hæmorrhage, so that I think we are justified in assuming that it does occur also in many of these cases of diplegia.

When the hæmorrhage is on both sides of the longitudinal fissure in such a way as to affect equally the motor areas for the legs, the usual diplegia results; in other cases it may be more extensive on one side than the other, and affect both legs and one arm; or it may be more extensive still, affecting all four limbs. The mental condition is often good, but it may not be so, and this is to be explained by extension of the hæmorrhage forwards beyond the limits of the motor cortex. Whether any intrauterine factor may also operate, or whether difficult labour is the sole cause, is not absolutely easy to say.

### Encephalitis.

Encephalitis may develop very rapidly or quite slowly. The resulting paralysis naturally depends on the distribution of the inflammation in the brain, but, as a matter of fact, the result is nearly always a diplegia. Local encephalitis may, however, give rise to hemiplegia, and this is a very definite clinical entity. The child is quite well until it suddenly has a convulsion or series of convulsions, after which hemiplegia, often followed by athetosis, is observed. The hemiplegia may persist throughout life, and Jacksonian fits are also common on the affected side. It has been thought by some observers that these fits begin in the unaffected limbs, but my own experience is that they are confined to the paralysed side. This form of encephalitis is found in children up to five or six years of age. It has been thought that the pathological condition is the same as that underlying acute anterior poliomyelitis in the spinal cord.

Acute ataxy is probably the result of a similar inflammation in the cerebellum.

In a certain number of cases children develop many of the signs and symptoms of general paralysis of the insane. They have often been abnormally bright and clever children, but at eleven or twelve years of age they begin to have fits, tremor, ataxia and difficulty in walking, irresponsibility, and other mental symptoms. In most of these cases the patient can be proved to be the subject of congenital syphilis, and, although the stigmata cannot always be found, the disease may be looked upon as almost certainly syphilitic in origin.

Juvenile tabes is a less well-marked condition, but it does occur. It seems to be especially associated with optic atrophy, which is relatively much commoner than in adults who have the same disease.

### Spinal Palsies.

Infantile paralysis, or acute anterior poliomyelitis, is by far the most important and characteristic of children's paralyses. It is beyond question an affection of the anterior horn cells of the cord, but the ultimate pathology is still unknown—that is, it is uncertain whether the determining cause of the thrombosis in the anterior spinal arteries is microbic or chemical in nature. It is certainly extraordinary that a microbe should be able to select certain vessels in this way, though not impossible, for there are drugs known which possess selective actions on parts of the nervous system, of which I need only mention atropine.

The mode of onset varies. Sometimes there is an acute feverish attack with pain. Sometimes a child goes to bed healthy and wakes up in the morning with a paralysed limb. In the latter class it is generally only one limb that is involved. In other cases there is a definite history of accident, but whether the paralysis is in any way determined by, or a reflex manifestation of, the accident is most difficult to say. I have seen so many cases in which there is a history of recent accident that I cannot refuse to believe that there is some connection between that and the paralysis.

There is always marked wasting seen in the affected limbs, which are thus distinguished from those of hemiplegics. Even in the latter after a few years there is evident a difference between the two sides, but this is due really to failure of normal growth rather than to wasting strictly so-called; for that which has never appeared cannot be said to have wasted away.

### Friedreich's Ataxia.

Friedreich's ataxia is a sclerotic condition of the posterior and lateral columns of the spinal cord, often affecting several members of the same family. The back is usually curved in a well-marked S-shaped manner, and there are also characteristic deformities of the feet. Ataxia and difficulty in walking are the most prominent features. A somewhat similar disease, though not a very definite clinical entity, is cerebellar ataxia. The cases resemble Friedreich's disease clinically, but differ in presenting exaggerated reflexes instead of absence of them. They also often show optic atrophy.

### Myopathies.

Although there are different groups of myopathies, the disease is really one and indivisible. Thus a case of pseudo-hypertrophic paralysis such as you have just seen may go on to a completely atrophic myopathy, which would, if seen for the first time, be diagnosed as idiopathic muscular atrophy. However, even when all the other muscles are wasted, it will often be found that the infra-spinatus still shows signs of pseudo-hypertrophy.

There is, besides these two kinds, yet another group of myopathies in which the facial and scapular muscles are especially affected. The orbiculares orbis and palpebrarum are atrophied, and the patient can then neither whistle nor shut his eyes. The names of Landouzy and Déjerine are associated especially with the study of this condition, but it has been pointed out that the symptoms may supervene in one of the other forms of myopathy, or the two may be associated. Moreover, in one family there may be examples at the same time of the Landouzy-Déjerine type, and of some other kind of myopathy. The striking point about all these myopathies is that no changes have been discovered in the spinal cord or any other part of the central nervous system, and strictly, therefore, these are not true nervous diseases, although they are generally dealt with in text-books of the nervous system. The peroneal type is another variety of the same disease, in which the muscles of the legs are wasted to an extreme degree: the atrophy spreads upwards to the thighs and ultimately to other parts of the body. In some cases the recti abdominis are especially affected, which results in an extraordinary bulging of the abdomen.

Meningitis is nearly always fatal, and I need do no more than mention it as a possible cause of paralysis in children. There are cases of meningitis that recover, and are then followed by various forms of paralysis; but as a cause of children's palsies meningitis is not of any great practical importance.

